# Influence of patient-related factors on intraoperative blood loss during double opposing Z-plasty Furlow palatoplasty and buccal fat pad coverage: A prospective study 

**DOI:** 10.4317/jced.59407

**Published:** 2022-08-01

**Authors:** Diandra-Sabrina Natsir-Kalla, Muhammad Ruslin, SA Alkaabi, Andi-Sitti-Hajrah Yusuf, Andi Tajrin, Tymour Forouzanfar, Siswanto, Hedi Kuswanto, Paolo Boffano, Lun-Jou Lo

**Affiliations:** 1 Amsterdam UMC and Academic Centre for Dentistry Amsterdam (ACTA), Vrije Universiteit Amsterdam, Department of Oral and Maxillofacial Surgery/Pathology, Amsterdam Movement Sciences, de Boelelaan 1117, Amsterdam, the Netherlands; 2 Department of Biochemistry, Faculty of Medicine, Hasanuddin University, Makassar, Indonesia; 3 Department of Oral and Maxillofacial Surgery, Faculty of Dentistry, Hasanuddin University, Makassar, Indonesia; 4 Department of Oral and Maxillofacial Surgery, Fujairah Hospital, Fujairah, Ministry of Health, Emirates Health Service; 5 Department of Statistics, Hasanuddin University, Makassar, Indonesia; 6 Division of Maxillofacial Surgery, “Maggiore della Carità” University Hospital, University of Eastern Piedmont, Novara, Italy; 7 Department of Plastic and Reconstructive Surgery, Craniofacial Research Center, Chang Gung Memorial Hospital, Chang Gung University, Taoyuan, Taiwan

## Abstract

**Background:**

Surgical procedures including palatoplasty have a risk for complications. The aim of this study was to investigate the intraoperative and early postoperative blood loss using the buccal fat pad (BFP) during cleft lip and/or cleft palate (CL/P) surgery.

**Material and Methods:**

This prospective study included a total of 109 patients with cleft palate (CP) during a three-month period of treatment at Hasanuddin University Dental Hospital (permanent center) and charity trips in rural parts of Eastern Indonesia. All patients were treated with DOZ Furlow technique combined with BFP graft. Before and after surgery, the total amount of intraoperative blood loss was calculated by measuring the weight differences of the gauze swabs that were used to control the surgical bleeding followed by a complete blood count at three days postoperatively.

**Results:**

The difference in the amount of blood loss based on age categories in charity groups was found to be significant (*P*<0.05). Overall, we found that high body weight and operation time significantly contributed to increased blood loss (*P*<0.05).

**Conclusions:**

Weight and operative time can contribute to more blood loss during palatoplasty.

** Key words:**Buccal fat pad, complication, cleft lip, cleft palate, palatoplasty.

## Introduction

Indonesia is among the countries with a high number of CL/P cases in which some of them left untreated specifically those who lived in the rural area (1,2). In the developing and low-middle income countries (LMC), a common model to provide cleft treatment in remote sites is through charity events (3). However, there are sometimes many barriers in presenting treatments for patients in this area including lack of safe operating facilities, lack of equipment, lack of well-trained surgeons, lack of associated specialists and anesthesia providers who can undertake the surgical treatments (4). Consequently, these various shortages cause the surgical capacity in the remote area to be generally inadequate (3).

In the course of CL/P repair, the surgery is usually carried out when the patients is within the first 12 months of life (5). At this age, patients with a body weight between 5 kg and 10 kg and a blood volume between 400 mL and 700 mL can have more blood loss, which should be taken seriously (5,6). Furthermore, when patients present at an older age, complex cleft may also result in a higher risk of bleeding (5). This patient’s characteristic was, in fact, commonly presented among patients with CL/P in Indonesia due to the limited healthcare setting that contributed in the treatment delay (2) .

While the topic of blood loss during palatoplasty is much discussed in the literature, there is not much consensus between these articles (5,7). What is missing in the literature is a study that discusses the relationship between intraoperative blood loss and patient-related factors. Therefore, this study aims to identify which factors affect the amount of blood loss during palatoplasty using the DOZ Furlow technique combined with BFP as graft materials.

## Material and Methods

-Study design and patient recruitment

This prospective study was approved by the ethical committee of Hasanuddin University Makassar, Indonesia. Informed consent was obtained from candidates and/or their parents or legal guardian who were willing to join the study after being fully educated about the procedure. Patient data were collected during a three-month period (March-May 2017) of five charity trips to different regions in Eastern Indonesia and at Hasanuddin University Dental Hospital. The team of the charity trips consisted of oral and maxillofacial surgeons, anesthesiologists, surgical assistants, general dentists, and medical or dental students. Hasanuddin University Dental Hospital is a secondary referral hospital staffed by a team of multidisciplinary cares.

-Sample size determination 

We firstly collected the number of patients in charity group and the number of patients in the hospital group were adjusted accordingly. The reason was because charity surgeries were conducted in limited settings that made it less adjusTable than hospital.

-Study inclusions and exclusions

Before starting with this study a number of inclusion and exclusion criteria were set up: patients with cleft palate which needed primary palatoplasty would be included in this study, while patients with syndromic clefts, fistula after palatoplasty which needed reconstruction, cleft after trauma, patients affected with multiple syndromes, and patients with a family history of blood loss conditions were excluded from this study. In addition, procedures using techniques to close palatal clefts other than the DOZ Furlow technique combined with BFP graft were also excluded.

-Operation technique

**Figure 1:**
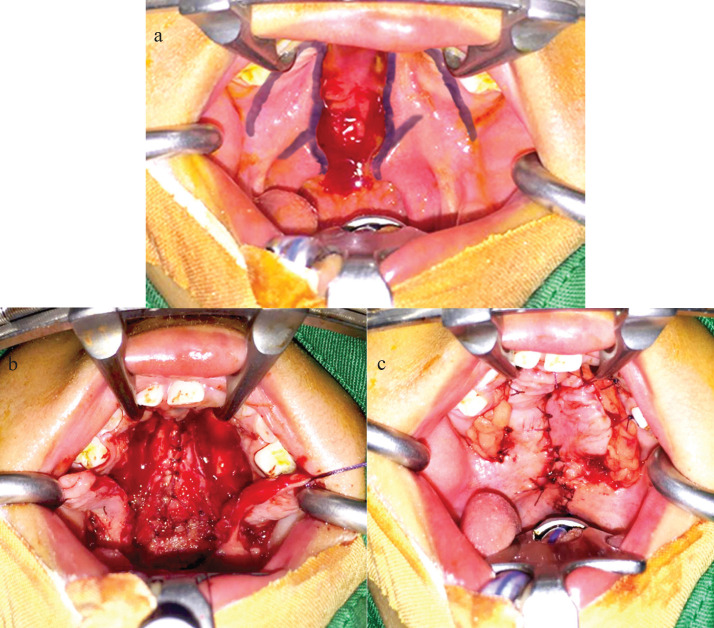
Operation procedure: (a) Flap design; (b) Bisection of mucosal and muscle layers, then suturing the nasal mucosa lining with the z-plasty technique; (c) Suturing the oral mucosal and placing the BFP towards the defect area with the interrupt technique.

The patients were put under general anesthesia and got prepared for surgery. The patients were injected with a local anesthetic solution of 2-5cc lidocaine 2% with epinephrine 1:100.000 alongside the line of incision (8). The first incision was made five minutes later and from that moment on, the operative time was measured. The operation procedure of cleft palate closure is depicted in Figure 1. At first, the oral flaps were created by making an incision alongside the margin of the cleft and then continuing into a relaxing incision alongside the processus alveolar and ending posteriorly at the hamular processes of the palatinal bone (9,10) (Fig. 1a). With a raspatorium, the mucosa was lifted of the hard palate and elevated with a thick suture, these mucoperiosteal flaps were used to close the hard palatal defect. To close the nasal cleft, the surgeon would make a mirrored DOZ-plasty: the nasal mucosal flap anteriorly and the nasal myomucosal flap posteriorly (Fig. 1b). The DOZ flaps on the nasal side were transposed and inset. Then the oral flaps were transposed and closed at midline (9,11). To prevent scarring and post-operative complications the surgeons transplanted the BFP into the open relaxing incisions (12,13) (Fig. 1c).

-Data collections

The intraoperative surgical form was completed with following data recorded: width of cleft, type of cleft, technique used, and operative time. Patient’s age was categorized in 4 groups according to the previous study with some adjustment: young child (<6 years), child (6-12 years), adolescent (12-18 years), and adult (>18 years) (14). The intraoperative width of the cleft was measured using a Castroviejo caliper (15) and the type of cleft was noted using the Veau classification (16).

Prior to the surgery, a blood count was performed as a preoperative screening method. During the surgery, intraoperative blood loss was calculated by measuring the difference in weight of the gauze swabs before and after surgery. The blood-soaked gauze swabs were collected in a metal container lined with a plastic bag to prevent vaporization. After surgery, the swabs would be weighed on an electrical analytical balance (PT. Kenko Electric Indonesia) and the results noted on the intraoperative form. The patients got admitted to the hospital postoperatively for three days and on the third day of their stay, another blood count would be taken from each patient to analyze any relationship between the amount of blood loss and values.

-Statistical analysis

The database was created on Microsoft Excel for Mac 2011 version 14.1.0. The statistical analysis was performed using IBM SPSS Statistics 24. In order to identify the difference of blood loss between charity trips and permanent hospital, nonparametric Mann Whitney test was used. To find the difference between the amount of blood loss from the two groups based on age category and cleft type, nonparametric Kruskal Wallis was done. To evaluate a potential relationship between the amount of blood loss and the numeric and categorical variables, the linear regression with backward method was used. P-values <0.05 were considered statistically significant.

-Ethical Consideration

Ethical approval (approval number: UH14060319) was granted by the Health Research Ethics Committee of the Faculty of Medicine, Hasanuddin University, Makassar, Indonesia. Because of the general age of the patients, the parents or legal guardian signed the research form if they would consent to the study.

## Results

The estimations of blood loss were made on a total of 109 patients. As seen in Table 1, a total of 50 patients (29 male and 21 female) were treated during charity trips (group 1) and 59 patients (26 male and 33 female) were treated in the permanent hospital (group 2). The mean age of patients in group 1 was 90.52 months (about 7 years), while the mean age of patients in group 2 was 118 months (about 9 years). The mean weight of the 50 cases was 22.94 kg and the mean weight of the 59 cases was 21.96 kg. The mean operation time in group 1 was 90.93 minutes (SD 38.72) and 94.48 minutes (SD 26.37) in group 2. No significant differences were found between the hemoglobin and hematocrit values before and 3 days after surgery.

**Table 1:**
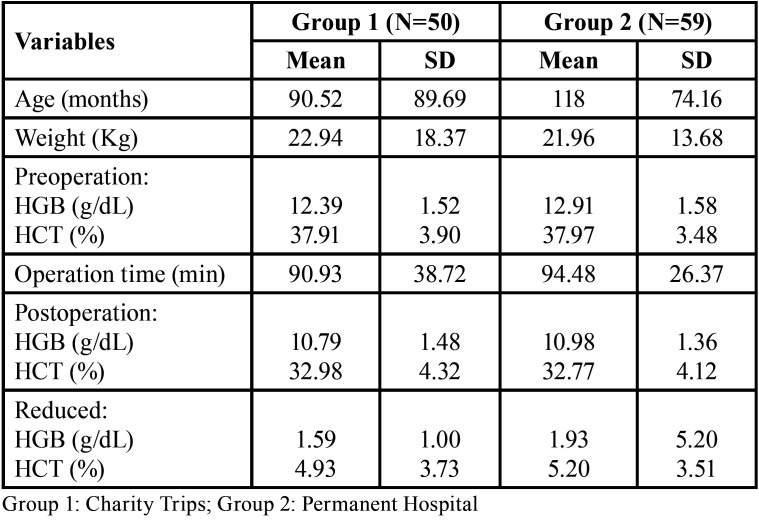
Patient characteristics in the pre-and post-operation period.

Table 2 highlights the comparison of the total amount of blood loss between patients treated during charity trips and permanent hospital. It was seen that the measured blood loss differed much in between patients. Even though the mean blood loss was lower in group 1 (98.69 mL) compared to group 2 (106.39 mL), there is no significant difference in the amount of blood loss between the two groups (*P* >0.05). Of the total included patients, only one patient who was treated during charity trips suffered a postoperative complication, i.e. active bleeding. On further inspection, it was due to a ruptured suture. None of the 109 patients needed any blood transfusion during or after surgery. Furthermore, in all surgeries, wound closure was performed with the use of BFP graft. No other techniques or additional relaxing incisions were used.

**Table 2:**
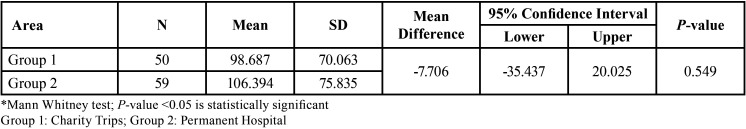
Comparison of the total amount of blood loss between the two groups.

Table 3 shows the results of the Kruskal Wallis test where we are looking at the difference between each age category and cleft type based on the amount of bleeding from the two groups. It was found that the difference between age categories is significant in group 1 (*P* <0.05). Thus, Pairwise Comparisons were done to investigate the partial difference in this category using superscript code. Based on the analysis, it can be said that there is a significant difference in the amount of blood loss between age categories of patients treated during charity trips. The category of young child has significantly lower blood loss compared to the child, adolescent, and adult.

**Table 3:**
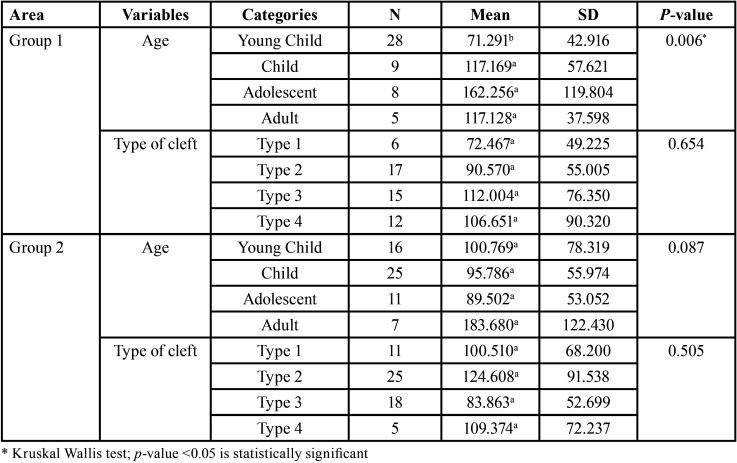
Difference between age categories and cleft types based on the total amount of blood loss (mL) from two groups.

To analyze patient-related factors that may influence the intraoperative bleeding during palatoplasty procedure in 109 subjects, a linear regression test has been performed. In this study, the independent variables are age, gender, weight, width of the gap, type of the cleft, operation time, and location of the surgery. From Table 4, it can be deduced that weight and operation time have significant effects (*P*<0.05) which means that the higher the weight and the longer the operation time are, the more at risk the patients are for increased blood loss during palatoplasty procedure.

**Table 4:**
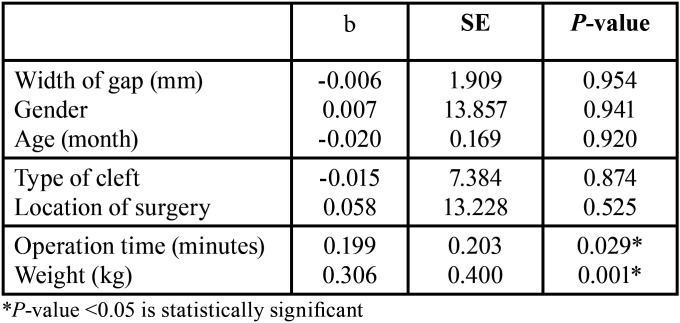
Regression linear test between variables and the amount of blood loss.

## Discussion

 The aim of this study was to identify patient-related factors that affect the amount of blood loss during palatoplasty using the DOZ Furlow technique in combination with BFP. To the best of our knowledge, previous studies have only discussed the amount of blood loss that can be expected during DOZ Furlow palatoplasty (5,17).

One hundred and nine CL/P patients were included in this study. Patients’ mean age were 90.52 months (±7 years) and 118 months (±9 years) for charity trips group and permanent hospital group respectively. In the previous study conducted by Katzel *et al*. (2009), palatoplasty is performed in patients aged between 6 and 12 months, resulting in satisfying outcomes (18) Our different age range is due to the fact that our study was performed in a developing country with a poorly developed health care system causing a late diagnosis on patients (2,19,20). Furthermore, this problem is more difficult because those living in rural areas do usually not have money to finance the surgery and extra travelling costs to where the treatment is provided (21–23). Adeyemo *et al*. also found that reasons for late CL/P repair specifically among rural populations were lack of awareness of treatment availability (13.3%), lack of health care services nearby (18.4%), and lack of money (56.7%) (20). This condition seems to be consistent with the condition in rural areas in Indonesia, thus, patients have to wait for a charity surgery at nearby village/city that further will delay the CL/P treatment (2). In contrast, it is a routine procedure in Western countries to prepare pre-and postnatal plans for infants with CL/P (18,24). Early counseling and treatment planning for CL/P patients were shown to have a better outcome in some aspects such as speech, cosmetic, and psychological perspectives (25,26).

In the present study, the mean blood loss was 98.69 mL and 106.39 mL for the charity trips and permanent hospital groups, respectively. These were relatively higher than previously published studies (5,17) . Kim *et al*. (2018) reported a mean blood loss of 16.61 mL, but their patients had lower mean age, lower mean weight, less severe mean cleft type, and smaller mean palatal gap than the patients in our study (5). We also found that older children (8-9 years) were at high risk for increased intraoperative blood loss during palatoplasty especially those performed in the rural areas. We have to take into account that the palate is a highly vascularized structure in the mouth with multiple vessels alongside its width and length that needs to be handled properly (27). Therefore, early assessments and surgical interventions to patients with CL/P are highly recommended.

Intraoperative time seemed to be correlated with the amount of blood loss in the present study. Longer operative time will result in higher amount of intraoperative blood loss. One contributing factor might be that the surgeries were performed by a team of surgeons and not all of them were experienced in performing palatoplasty using DOZ technique with BFP graft. Nevertheless, no blood transfusions were deemed necessary to any of the patients because pre-operative screening was performed according to the Practice Guidelines for perioperative blood management (2015) as predictor of perioperative blood loss, risk of transfusion, or other adverse events associated with transfusion(28). During this screening, the anesthesiologist looked at the blood values and excluded patients with any blood condition present. High amounts of blood loss were also not expected during surgeries because of the procedure type(5,17). The relatively low intraoperative blood loss as determined in this study showed that DOZ palatoplasty is a relatively safe procedure.

This study has several limitations. First, the small sample size prohibited adjudication of the found statistical significance. Secondly, a difficult point of this study was to measure blood loss as accurately as possible. A previous study by Daabiss *et al*. measured the blood loss by using a visual comparative colorimeter (29) which is not applicable in the limited clinical setting of rural areas in Indonesia. In addition, this method is particularly suiTable for large amounts of blood loss unlike the expected low blood loss from palatoplasty procedures. In this study, the amount of blood loss was measured by using the weight difference between clean and blood-soaked gauze swabs. The average density of human blood is known to be close to pure water so that weight in mg can be converted into mL (5,17). This method, however, might still underestimate the amount of blood loss because of technical reasons such as a surgeon accidentally using the suction system, or the inability to measure the amount of blood loss left on the instruments, gloves, and surgical drapes. Postoperative blood loss was also not measured because the present clinical setting prohibited that to be performed. Nevertheless, we still think that the last point was not detrimental to the study because our focus was on the intraoperative blood loss during palatoplasty.

## Conclusions

Our results suggest that DOZ Furlow palatoplasty combined with BFP graft is a relatively safe procedure. This study found that the procedure resulted in minimal to mild amounts of blood loss, however, higher weight and longer operation time caused significantly more blood loss. The first recommendation from this study is to operate patients at an earlier age. Operating on young children does not only reduce the amount of intraoperative blood loss but also gives the patients a better start at life. Our second recommendation is to shorten the operation time as much as possible since this will significantly decrease the amount of intraoperative blood loss. Furthermore, standardized postnatal holistic planning is recommended in Indonesia for the improvement of cleft care.
